# Diet is not the primary driver of bacterial community structure in the gut of litter-feeding cockroaches

**DOI:** 10.1186/s12866-019-1601-9

**Published:** 2019-10-30

**Authors:** Niclas Lampert, Aram Mikaelyan, Andreas Brune

**Affiliations:** 1 Research Group Insect Gut Microbiology and Symbiosis, Max Planck Institute for Terrestrial Microbiology, Karl-von-Frisch-Str. 10, 35043 Marburg, Germany; 2 Present Address: Department of Entomology and Plant Pathology, North Carolina State University, Raleigh, NC 27607 USA

**Keywords:** Insects, Gut microbiota, Deep sequencing, Cockroaches, Lignocellulose

## Abstract

**Background:**

Diet is a major determinant of bacterial community structure in termite guts, but evidence of its importance in the closely related cockroaches is conflicting. Here, we investigated the ecological drivers of the bacterial gut microbiota in cockroaches that feed on lignocellulosic leaf litter.

**Results:**

The physicochemical conditions determined with microsensors in the guts of *Ergaula capucina*, *Pycnoscelus surinamensis*, and *Byrsotria rothi* were similar to those reported for both wood-feeding and omnivorous cockroaches. All gut compartments were anoxic at the center and showed a slightly acidic to neutral pH and variable but slightly reducing conditions. Hydrogen accumulated only in the crop of *B. rothi*. High-throughput amplicon sequencing of bacterial 16S rRNA genes documented that community structure in individual gut compartments correlated strongly with the respective microenvironmental conditions. A comparison of the hindgut microbiota of cockroaches and termites from different feeding groups revealed that the vast majority of the core taxa in cockroaches with a lignocellulosic diet were present also in omnivorous cockroaches but absent in wood-feeding higher termites.

**Conclusion:**

Our results indicate that diet is not the primary driver of bacterial community structure in the gut of wood- and litter-feeding cockroaches. The high similarity to the gut microbiota of omnivorous cockroaches suggests that the dietary components that are actually digested do not differ fundamentally between feeding groups.

## Background

Cockroaches are the closest relatives of termites [1, 2]. The intestinal tracts of both insect groups are densely colonized by a symbiotic gut microbiota of bacteria and archaea, and sometimes also unicellular eukaryotes [3–5]. The gut microbiota of termites and its role in symbiotic digestion have been studied intensively during the past decades (for reviews, see [6–8]). In all evolutionarily lower termite families, lignocellulose digestion is carried out primarily by a dense assemblage of symbiotic flagellates, which are absent in all cockroaches and higher termites (family Termitidae). In the wood-feeding members of the Termitidae, their key roles in the digestion of cellulose and hemicelluloses were apparently replaced by specific lineages of *Fibrobacteres* and *Spirochaetes* [9–11].

Much less is known about the bacteria colonizing the intestinal tracts of cockroaches and their role in symbiotic digestion. While termites are highly specialized on a lignocellulosic diet, cockroaches are mostly omnivorous scavengers that typically exploit a variety of food sources [12]. Nevertheless, lignocellulosic plant litter and decaying wood present a major food source for many species, and lignocellulose digestion by cockroaches is considered to play a critical role in the turnover of organic matter in forest ecosystems [13].

In the wood-feeding *Parasphaeria boleiriana* (Blaberidae: Zetoborinae) and all members of the genera *Panesthia* and *Salganea* (Blaberidae: Panesthiinae), which dwell in decaying wood logs [13–15], xylophagy most likely evolved independently from that in the termite clade [14]. Also many detritivorous cockroaches feed on leaf litter or other diets rich in lignocellulosic substrates [16]. The survival of xylophagous Panesthiinae on pure cellulose has been attributed to the presence of glycoside hydrolases produced by both the host and its gut microbiota ([15, 17]; for a review, see [18]), but detailed balances of plant polymer degradation in litter-feeding cockroaches are lacking.

Next to diet, niche heterogeneity has been recognized as another important determinant of bacterial community structure in the hindgut of termites. The composition of the termite gut microbiota is not only characteristic for members of different feeding groups [19] but also differs among the individual compartments of their intestinal tract [20] and between microhabitats located within the same compartment, such as gut wall, fiber fraction and luminal content [10, 21]. These differences in community structure are usually accompanied by changes in both microenvironmental conditions (pH, oxygen status, and intestinal redox potential) and microbial activities [22–25].

In all cockroaches investigated to date, microenvironmental conditions are rather uniform. The gut content is slightly acidic to neutral and has a negative redox potential [26–28]. In adult cockroaches, the center of all gut compartments is typically anoxic, but in the gut of early larval stages, suboxic conditions have an impact on microbial community assembly during host development [29]. Hydrogen accumulation has been reported only for the posterior midgut of the omnivorous scavengers *Blaberus* sp. and *Shelfordella lateralis* (maintained on formulated rabbit or chicken feed) [26, 30], and for the crop of *Panesthia angustipennis* (maintained on decaying wood) [27]. Each major gut compartment of the omnivorous *S. lateralis*, the wood-feeding *P. angustipennis*, and a detritivorous *Panchlora* sp. (maintained on refuse pile material of leaf-cutter ants) distinctly differs in structure and composition of its bacterial community [26, 27, 31]. In experiments with germ-free *S. lateralis* that were inoculated with gut communities from various hosts, similar microbial lineages were selected by the gut environment, irrespective of the inoculum [32], which suggests a strong selection pressure by the microenvironmental conditions and the functional niches available in the gut.

It remains unclear whether structure and composition of the bacterial gut microbiota of cockroaches are strongly affected by diet. A significant response of the hindgut microbiota to diets with different protein contents was found in the omnivorous *Blattella germanica* [33] but contrasts with a resilience to dietary changes reported for *Periplaneta americana* [34]. In *S. lateralis*, potential effects of high-protein and high-fiber diets of bacterial community structure were masked by strong individual variations [35]. The high similarity in the bacterial community structures of omnivorous cockroaches and a *Panchlora* sp. that lives in the refuse piles of fungus-cultivating leafcutter ants suggests the existence of a core microbial community that is independent of a particular diet [31]. However, the number of cockroach species investigated so far is too small to test the effects of host diet on bacterial community structure, and information on representatives that thrive on lignocellulosic plant litter is sorely needed.

We addressed this gap by characterizing the bacterial gut microbiota of cockroaches from the genera *Byrsotria*, *Pycnoscelus*, and *Ergaula*, which represent litter feeders from three subfamilies (Blaberinae, Corydiinae, Pycnoscelinae), are available from commercial breeders, and can be maintained on a diet of dried oak leaves. Using high-throughput amplicon sequencing of the bacterial 16S rRNA genes, we taxonomically analyzed the communities using a phylogenetically curated reference database (DictDb), tailor-made for the accurate identification of bacterial lineages specific to termite and cockroach guts [36], and compared community structure and composition to previously published datasets of cockroaches from other diet groups. To identify differences in microenvironmental conditions responsible for differences in community structure between compartments, we used microsensors to determine oxygen and hydrogen partial pressure, intestinal pH, and redox potential of the gut lumen along the entire intestinal tract. To determine whether host diet determines bacterial community structure in cockroaches, we identified the core bacterial families in cockroaches with a lignocellulosic diet and compared them to those in omnivorous cockroaches and xylophagous higher termites.

## Results

### Physicochemical conditions in different gut compartments

**Fig. 1:**
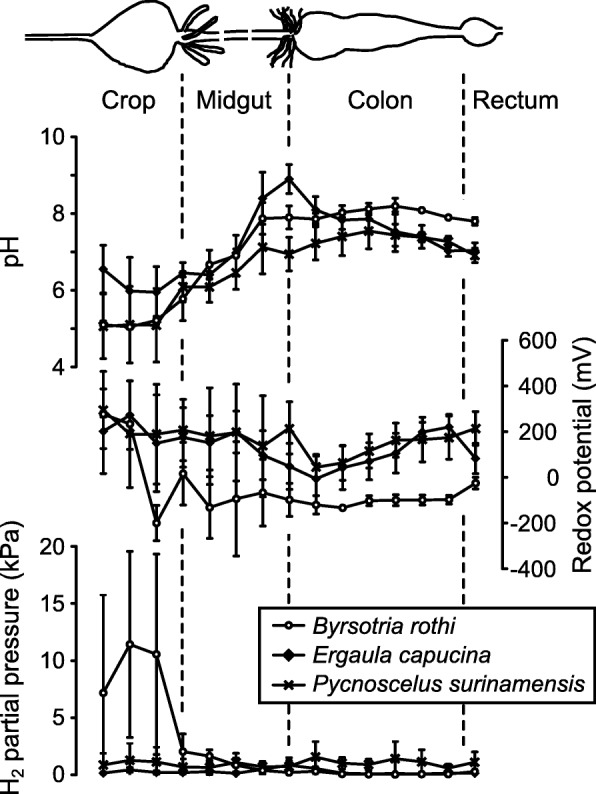
Axial profiles of intestinal pH, redox potential, and hydrogen partial pressure in the gut of litter-feeding cockroaches, determined with microsensors. Note that the gut axis has been normalized, i.e., the distances between the points of measurement are not absolute but instead represent cardinal points of each gut compartment (e.g., anterior, median, and posterior crop). In reality, the midgut region is considerably longer. Average length of the extended gut was estimated to be 84 mm (*Byrsotria rothi*), 46 mm (*Ergaula capucina*), and 33 mm (*Pycnoscelus surinamensis*) using a ruler. All microsensor measurements were made at the gut center; symbols indicate means with standard error of three guts

We obtained axial profiles of pH, redox potential, and hydrogen partial pressure in the intestinal tracts of *Byrsotria rothi*, *Ergaula capucina,* and *Pycnoscelus surinamensis* (Fig. 1). In *B. rothi* and *P. surinamensis*, the pH was acidic in the crop (pH 5.1 ± 0.1 and 5.1 ± 0.9, respectively) and increased steadily along the midgut to neutral or slightly alkaline values in the hindgut (pH 8.0 ± 0.1 in *B. rothi*, and pH 7.4 ± 0.3 in *P. surinamensis*). In *E. capucina*, the crop was significantly less acidic (pH 6.2 ± 0.7); the pH showed a distinct alkaline maximum (pH 8.9 ± 0.4) at the midgut/hindgut junction and decreased again to neutral in the posterior hindgut.

The redox potential of the gut contents, measured at the gut center, was highly variable in crop and midgut but more consistent in the hindgut compartment of all species. Although all compartments were anoxic at the gut center (not shown), negative redox potentials (− 100 to − 200 mV) were observed only in *B. rothi*. In the other species, the values ranged from + 100 to + 200 mV, even in the dilated hindgut. Hydrogen partial pressure was either low (0.3–3.5 kPa) or below the detection limit (< 0.1 kPa in the hindguts of *B. rothi*). Only *B. rothi* showed a moderate accumulation of hydrogen in the crop (6–21 kPa). Oxygen partial pressure was below the detection limit at the center of all compartments (not shown).

### Community structure of homologous gut compartments

Amplicon sequencing of the V3-V4 region of the bacterial 16S rRNA genes in crop, midgut, and hindgut of the three cockroach species yielded between 60,000 and 170,000 high-quality sequence reads per sample (Table 1). We identified a total of 4297 OTUs (at 97% sequence similarity), with 800 to 1200 OTUs per sample (Table 1); rarefaction analysis of each sample indicated that 99.3–99.7% of the expected OTUs were recovered and sequencing depth was sufficient for all samples (Additional file 1: Figure S1). The number of OTUs recovered and the diversity and evenness of the respective communities were always higher in the hindgut samples. Except for the samples from *E. capucina*, > 99% of the reads were assigned at the phylum level. In all samples, classification success was high at class (> 97%) and family (> 72%) levels. At the genus level, a high classification success (> 64%) was achieved only in the hindgut compartment; values dropped considerably in crop and midgut, which indicates that many genus-level lineages in these compartments were not represented in the reference database.

**Table 1:** Properties of the iTag libraries of the individual gut compartments obtained from different host species. Diversity indices for crop (C), midgut (M), and hindgut (H) are based on OTUs, classification success is based on the number of assigned reads at different taxonomic levels

Host species	Sample	Reads	OTUs (97%)	Diversity indices^a^			Classification success (%)				Acc. no.^b^
				Richness	Diversity	Evenness	Class	Order	Family	Genus	
*Ergaula capucina*	C	169,596	1116	1520	4.10	0.584	99.3	97.0	78.5	47.5	9604
	M	114,698	1166	1503	3.64	0.516	98.1	95.2	77.8	47.2	9602
	H	53,896	1515	1583	5.78	0.789	97.4	95.4	89.0	64.4	9603
*Byrsotria fumigata*	C	193,791	905	1422	3.45	0.506	99.5	98.4	72.3	41.7	9607
	M	68,113	810	1012	3.66	0.547	99.0	96.9	76.5	50.1	9605
	H	58,848	1437	1521	5.31	0.730	98.6	95.7	84.2	64.4	9606
*Pycnoscelus surinamensis*	C	170,089	1080	1375	3.28	0.469	98.8	98.1	86.6	56.8	9601
	M	100,215	1076	1405	3.05	0.437	99.3	97.8	89.9	77.0	9599
	H	151,774	1284	1669	4.88	0.681	99.6	98.1	92.3	73.2	9600

^a^Based on OTUs. Richness, Chao1 estimator [37]; diversity, nonparametric Shannon index [38]; evenness index [39]

^b^Genbank biosample accession number: SAMN0884*nnnn*

**Fig. 2:**
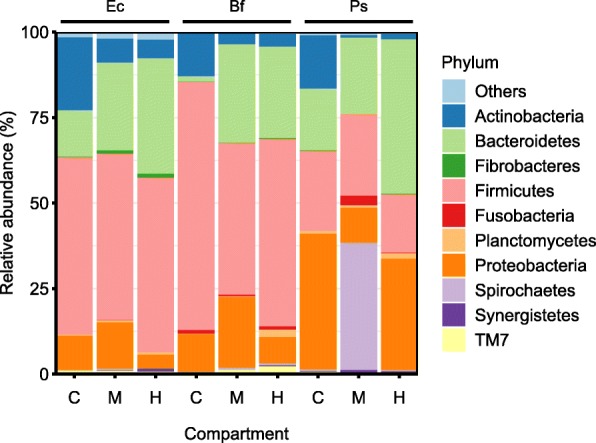
Relative abundance of bacterial phyla in the crop (C), midgut (M), and hindgut (H) of *Ergaula capucina* (Ec), *Byrsotria fumigata* (Bf), and *Pycnoscelus surinamensis* (Ps) fed on oak leaf litter. For details, see Additional file 2: Table S1

The bacterial communities in all samples comprised representatives from 28 phyla defined in the DictDb taxonomy. They were dominated (on average) by *Firmicutes* (43%), *Bacteroidetes* (24%), *Proteobacteria* (17%), and *Actinobacteria* (8%) (Fig. 2). *Actinobacteria* abundance peaked in the crop, whereas *Bacteroidetes* increased in abundance from crop to midgut to hindgut. In *E. capucina*, midgut and hindgut compartments contained small populations of *Fibrobacteres* (1%). The crop communities were dominated (on average) by lineages of *Bifidobacteriaceae*, *Lactobacillaceae*, *Lachnospiraceae* (all *Firmicutes*), and *Pseudomonadaceae* (*Proteobacteria*), which together represented more than one-third of the reads. By contrast, hindgut communities were dominated by *Porphyromonadaceae* and *Rikenellaceae* (both *Bacteroidetes*) and by *Lachnospiraceae* and *Ruminococcaceae* (both *Firmicutes*), and accounted (on average) for roughly two-thirds of the reads (Additional file 2: Table S1).

**Fig. 3:**
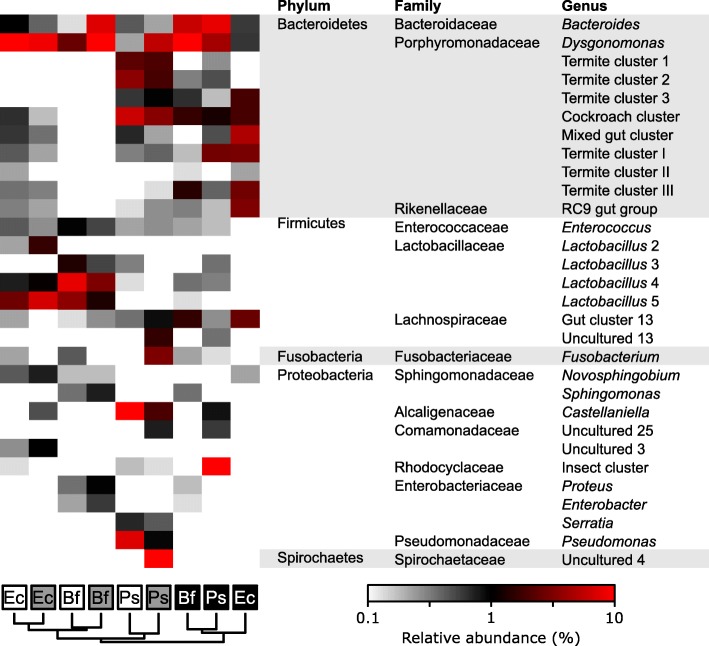
Relative abundance of the 30 most abundant genus-level groups in the crop (white), midgut (gray), and hindgut (black) of *Ergaula capucina* (Ec), *Byrsotria fumigata* (Bf), and *Pycnoscelus surinamensis* (Ps) fed on oak leaf litter. Phylogram indicates hierarchical cluster analysis of all classified reads (*hclust*, Euclidian distances). For numerical values, see Additional file 2: Table S1

The 30 most abundant genus-level groups differed in relative abundance between gut compartments (Fig. 3). For instance, *Bacteroides* (0.1–8.6%) and *Dysgonomonas* (0.1–18.3%) species were present in all gut compartments of the three hosts. While several *Lactobacillus* species and one *Enterococcus* species were consistently found in high abundance in the crop and midgut, the hindgut harbored mostly representatives of *Bacteroidaceae*, *Porphyromonadaceae*, and *Lachnospiraceae*, many of which remained unclassified at the genus level. *Pycnoscelus surinamensis* presented an exception to this trend; in this case, lineages such as *Castellaniella* and *Pseudomonas* in the crop, uncultured *Spirochaetaceae* in the midgut, and uncultured *Rhodocyclaceae* in the hindgut made up a major part of the bacterial community in the respective compartments.

**Fig. 4:**
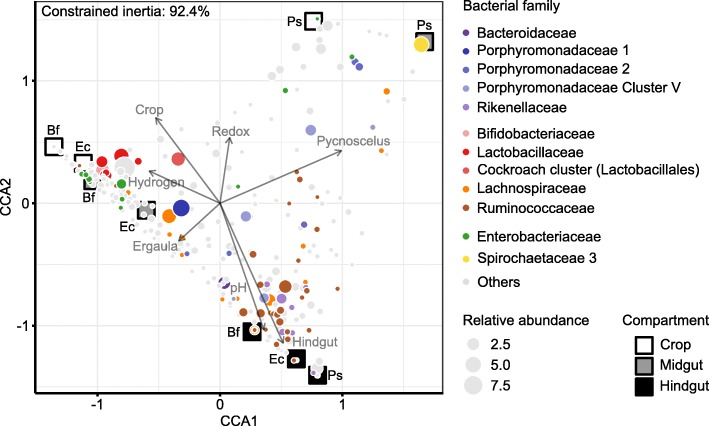
Canonical correspondence analysis (CCA) of the relative abundance of bacterial genera and environmental variables in gut compartments of the litter-feeding cockroaches *Ergaula capucina* (Ec), *Byrsotria fumigata* (Bf), and *Pycnoscelus surinamensis* (Ps). Each dot represents a genus-level group, with the color indicating the family affiliation and the size indicating its mean relative abundance. Each of the 435 bacterial genus-level groups was tested for covariance with the environmental variables: physicochemical conditions (pH, hydrogen partial pressure, and redox potential), host species, and gut compartment (gray labels). Approximate weighted averages of the communities in each gut compartment are shown as boxes labeled with the corresponding species abbreviation. Environmental variables are shown as directional axes (arrow length proportional to the total variance constrained by the variable). The position of a bacterial genus or community relative to the axis of an environmental variable indicates the level of correspondence between the respective genus or community and the environmental variable. Constrained inertia is equivalent to the total variance constrained by all environmental variables combined. For more details, see Additional file 2: Table S2

A canonical correspondence analysis (CCA) of bacterial community structure, physicochemical gut conditions, compartment, and host species revealed that these environmental variables constrained 92.4% of the variance in bacterial community structure (Fig. 4). The variables with the highest impact were the intestinal pH and the hindgut compartment, which corresponded significantly with changes in gut community composition (Additional file 2: Table S2). In that context, it is of interest that several bacterial lineages, most notably *Ruminococcaceae*, *Rikenellaceae*, and *Porphyromonadaceae*, were typically associated with the hindgut compartment, high pH, and low hydrogen partial pressure. Contrastingly, lineages such as *Lactobacillaceae* and *Enterobacteriaceae* corresponded with lower pH and higher hydrogen partial pressure. The crop and midgut of *P. surinamensis* hosted high numbers of *Pseudomonadaceae* and *Spirochaetaceae*, respectively, in association with high redox potential in both samples.

**Fig. 5:**
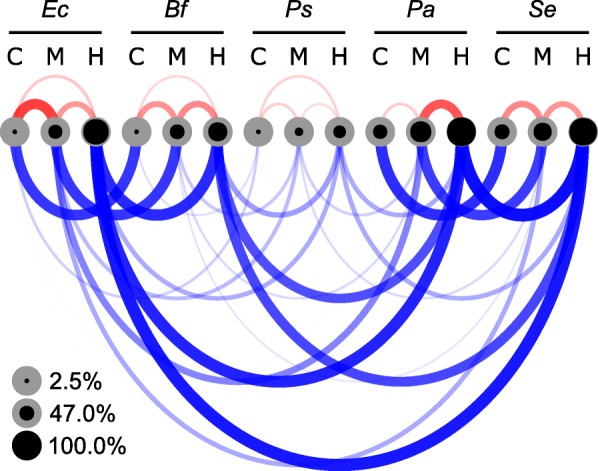
Similarity of the bacterial communities (family level) and abundance of core lineages in the different gut compartments of five lignocellulose-feeding cockroaches. Community similarity (Morisita-Horn index) between consecutive gut compartments of the same species (red) and between homologous gut compartments of different species (blue) is indicated by the width and the opacity of the connecting arcs. The relative abundance of core lineages (families represented in all homologous gut compartments) is indicated by the size of the concentric filling (black) of the circles, which represent the crop (C), midgut (M), and hindgut (H) compartments of *Ergaula capucina* (Ec), *Byrsotria fumigata* (Bf), *Pycnoscelus surinamensis* (Ps), *Panesthia angustipennis* (Pa), and *Salganea esakii* (Se). For numerical values, see Additional file 2: Table S3

A comparison of the five lignocellulose-feeding cockroaches revealed that the core families shared between homologous guts made up the bulk of the bacterial community in the hindgut compartments. The similarity at the family level between the homologous gut compartments of both wood- and litter-feeding hosts was much higher than the similarity between the different gut compartments of the same species (Fig. 5). *Pycnoscelus surinamensis* was an exception to this trend because the core communities shared with other cockroaches was very small. In all hosts, the average contribution of the core families to the entire bacterial community increased from crop (37%) to midgut (66%) to hindgut (81%).

Several core bacterial families made up a major part of the bacterial communities, especially in the hindgut. Here, the relative abundance of 18 core bacterial families (Additional file 2: Table S3) ranged between 46.0% (*P. surinamensis*) and 98.4% (*Panesthia angustipennis*). The different lineages of the polyphyletic *Porphyromonadaceae* together comprised the most abundant bacterial family in both the midgut and hindgut of lignocellulose-feeding cockroaches, covering on average 22 and 23% of the bacterial community, respectively. However, *Porphyromonadaceae*_1 were more abundant in the midgut, while *Porphyromonadaceae*_2, as well as previously undescribed members binned to *Porphyromonadaceae*, “Cluster V”, and “Gut group” were more abundant in the hindgut. More lineages that accounted for 22% of the bacterial hindgut community fell within the *Ruminococcaceae*, most of which had no cultured representatives (e.g., “insect cluster”, “gut cluster” and “uncultured”). Members of the genus *Ruminococcus* were abundantly represented in the hindgut of *Ergaula capucina* and *Byrsotria fumigata*, while *Papillibacter* was present in all hindguts except *Pycnoscelus surinamensis*. *Lachnospiraceae* made up on average 12 and 13% of the bacterial community in the crop and midgut of the lignocellulose feeders. In the hindgut, they were represented by several major lineages, such as sequences from “gut cluster 13” (without cultured representatives but related to *Butyrivibrio crossotus*) or “Incertae sedis 30” (with the cultured representative *Clostridium phytofermentans*). Large fractions of the midgut community of *Panesthia angustipennis* (17%) and *Salganea esakii* (10%) were made up of Ca. Arthromitus. *Endomicrobiaceae* were found in very low abundance (≤0.8%) in the hindgut communities of all lignocellulose feeders.

### Effect of diet on gut community structure

**Fig. 6:**
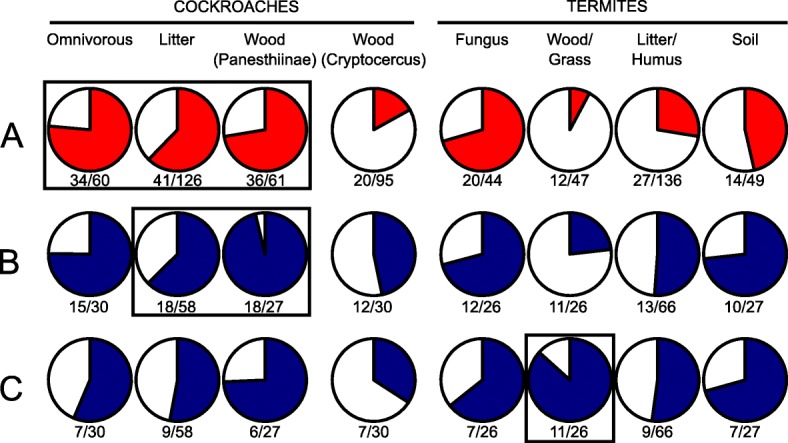
Core bacterial taxa in the hindgut of different feeding guilds of cockroaches and higher termites. The pie charts represent the average proportion of reads from core bacterial genera (red) and families (blue) relative to the entire bacterial community. The host groups used to define the respective core taxa are circumscribed with a rectangle: (**a**) all cockroaches except *Cryptocercus punctulatus*, (**b**) wood and litter-feeding cockroaches, and (**c**) wood-feeding higher termites. A core taxon was a genus present in > 70% or a family present in all members of the host groups in the respective rectangle. Numbers below the charts provide the average proportion of core taxa over the total number of taxa. For details, see Additional file 2: Table S3

To evaluate the impact of host diet on community structure, we determined the proportion of bacterial core taxa in different feeding groups of cockroaches and their representation in different feeding groups of higher termites, using data from this and previous studies (Additional file 2: Table S4). Lower termites were excluded from the analysis because their bacterial gut microbiota is strongly affected by their symbiotic flagellates [11]. First, we identified the core microbiota of cockroaches, disregarding the wood-feeding *Cryptocercus punctulatus*, whose gut microbiota is dominated by eukaryotic symbionts and resembles that of lower termites [11]. The majority of the bacterial community in all cockroaches (on average, 72% of the reads), irrespective of feeding group, consisted of core genera (genera presented in at least 70% of all host species) (Fig. 6a). By contrast, these core genera represented a much smaller proportion of the bacterial community in termites, with the fungus-feeding Macrotermitinae forming a notable exception. In wood-feeding higher termites, the core genera from cockroaches represented only 8% of the bacterial community.

To account for the tendency of members of the same bacterial family to carry out similar metabolic processes, and to rule out the possibility that bacterial lineages co-evolving with both termites and cockroaches were too dissimilar to be captured at the genus level, we extended the core taxon analysis to the family level. Most of the 18 bacterial families that were consistently represented in cockroaches with a lignocellulosic diet (i.e., wood- and litter-feeding cockroaches) were represented also in all omnivorous cockroach species (Fig. 6b). On average, the members of these families made up more than 90% of the bacterial community in the wood-feeding Panesthiinae, 60% in litter-feeding cockroach species, and 77% in omnivorous cockroach species. Their high relative abundance of core families from cockroaches in fungus-feeding termites was to be expected, but their representation in the other feeding groups was substantially higher than at the genus level. Again, the lowest proportion of reads assigned to bacterial core families from cockroaches was observed in the bacterial community in wood-feeding termites. Although their relative abundance was highest in fungus- or soil-feeding termite species, 13 out of the 18 core families present in wood- and litter-feeding cockroaches were represented also in litter- or humus-feeding termites, more than in any other termite feeding group. Litter-feeding cockroaches and litter- or humus-feeding termites were also similar in terms of taxon richness, i.e., the total number of bacterial genera (126 and 136) and families (58 and 66) represented in the respective communities, which were higher than in any of the other feeding groups. The relative abundance of the lignocellulose-feeding cockroach core families across all cockroaches ranged from 63 to 96%; within the cockroaches, the relative abundance was lowest in the litter-feeding cockroaches.

When we tested the representation of the 11 bacterial families that made up the core community in wood-feeding termites, we found that between six and nine of them were present also in cockroaches of all feeding groups (Fig. 6c). However, they represented a much smaller part of the total diversity and relative abundance of the respective communities.

Overall, the hindgut bacterial communities of cockroaches with a lignocellulosic diet featured core bacterial taxa different from those of wood-feeding termites. In some cases, similar core patterns on the family level between the different host feeding groups were due to the abundance of different genus-level lineages within the same family. For instance, *Lachnospiraceae* contributed, on average, 13 and 25% of the bacterial community in lignocellulose-feeding cockroaches and soil-feeding termites, respectively. However, while the undescribed “Gut cluster 13” within this family was among the most dominant genus-level groups in both host groups, soil-feeding termites additionally featured Ca. Arthromitus in high relative abundance (Additional file 2: Table S4). The *Rikenellaceae* were represented by *Alistipes* II in fungus-feeding termites, *Alistipes* IV in lignocellulose-feeding cockroaches, and *Alistipes* III and IV in omnivorous cockroaches. Notably, the *Acholeplasmataceae*, represented in most cockroaches by the genus *Acholeplasma* with up to 1.4% of the bacterial community, was completely absent in all higher termites and *Cryptocercus punctulatus*.

**Fig. 7:**
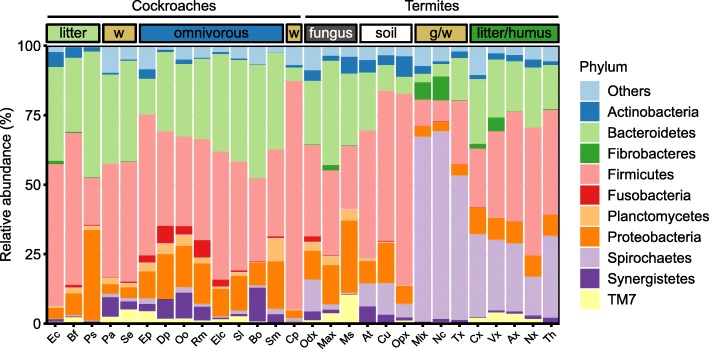
Relative abundance of major bacterial phyla in the hindgut communities of cockroaches and termites from different feeding groups (w, wood; g/w, grass/wood). Phyla with a mean relative abundance < 0.7% are summarized as “Others”. Host species are *Ergaula capucina* (Ec), *Byrsotria fumigata* (Bf), *Pycnoscelus surinamensis* (Ps), *Panesthia angustipennis* (Pa), *Salganea esakii* (Se), *Eublaberus posticus* (Ep), *Diploptera punctata* (Dp), *Opisthoplatia orientalis* (Oo), *Rhyparobia maderae* (Rm), *Elliptorhina chopardi* (Elc), *Shelfordella lateralis* (Sl), *Blatta orientalis* (Bo), *Symploce macroptera* (Sm), *Cryptocercus punctulatus* (Cp), *Odontotermes* sp. (Odx), *Macrotermes* sp. (Max), *Macrotermes subhyalinus* (Ms), *Alyscotermes trestus* (At), *Cubitermes ugandensis* (Cu), *Ophiotermes* sp. (Opx), *Microcerotermes* sp. (Mix), *Nasutitermes corniger* (Nc), *Trinervitermes* sp. (Tx), *Cornitermes* sp. (Cx), *Velocitermes* sp. (Vx), *Atlantitermes* sp. (Ax), *Neocapritermes* sp. (Nx), and *Termes hospes* (Th). Data from this and previous studies [19, 27, 11]; for details, see Additional file 2: Table S1

**Fig. 8:**
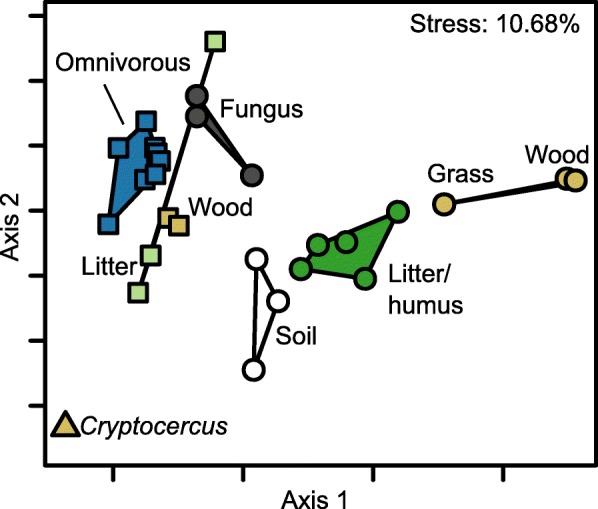
Similarity between the hindgut microbiota of cockroaches and higher termites, based on the weighted UniFrac metric and visualized by non-metric multidimensional scaling (NMDS). Polygons circumscribe cockroach species (squares) and termite species (circles) of the same diet groups (indicated by different colors). The wood-feeding cockroaches are *Panesthia angustipennis* and *Salganea esakii*; the wood-feeding *Cryptocercus punctulatus* (triangle), whose gut microbiota is dominated by eukaryotic symbionts and resembles that of lower termites, was treated as a separate group (see text). Species and color code for diet are the same as in Fig. 7

A comparison of community composition in the hindgut of wood- and litter-feeding cockroaches to those of other cockroaches and higher termites revealed major patterns between host groups that were apparent already at the phylum level (Fig. 7). Overall, the hindgut communities of cockroaches were clearly distinct in community structure from those of higher termites. A detailed comparison of the bacterial community structure based on the weighted UniFrac metric (Fig. 8) revealed that the hindgut communities of the omnivorous species were distinct from those of the wood- and litter-feeding species, which also displayed a higher degree of variation. Notably, the gut microbiota of the wood-feeding *Panesthia angustipennis* and *Salganea esakii* was quite dissimilar from that of the wood-feeding *Cryptocercus punctulatus*, confirming the affinity between all species harboring cellulolytic flagellates, whose abundant bacterial symbionts predominate the bacterial communities in their hindguts [11].

**Fig. 9:**
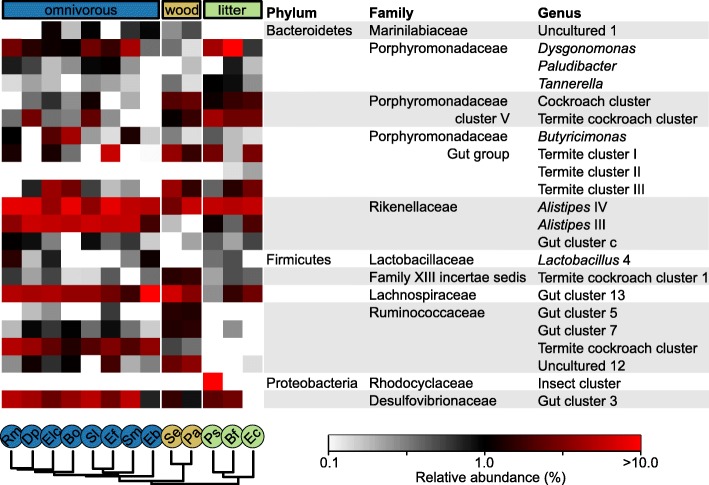
Heat map of the 22 most abundant bacterial genus-level groups in the hindgut of omnivorous (blue), wood (brown)- and litter-feeding (green) cockroaches. Hosts are *Rhyparobia maderae* (Rm), *Diploptera punctata* (Dp), *Elliptorhina chopardi* (Elc), *Blatta orientalis* (Bo), *Shelfordella lateralis* (Sl), *Eurycotis floridana* (Ef), *Symploce macroptera* (Sm), *Eublaberus posticus* (Eb), *Salganea esakii* (Se), *Panesthia angustipennis* (Pa), *Pycnoscelus surinamensis* (Ps), *Byrsotria fumigata* (Bf), and *Ergaula capucina* (Ec)

The majority of the reads obtained from the hindgut communities of cockroaches were assigned to *Firmicutes* and *Bacteroidetes*. Closer inspection revealed that many genus-level groups were shared between all cockroach species (Fig. 9). Among *Bacteroidetes*, the shared lineages include the genera *Dysgonomonas*, *Butyricimonas*, *Paludibacter*, and *Tannerella* (all *Porphyromonadaceae*). The core lineages with the highest relative abundance across all cockroach hindguts were found in the radiation of the super-genus *Alistipes* (*Rikenellaceae*); these core lineages were universally present in all samples. Their total abundance ranged from 2.7 to 18.2% of the reads obtained from the respective hosts. Other lineages present in all cockroaches include the so far uncultured “Gut cluster 13” (*Lachnospiraceae*). Among the few lineages that were specifically enriched in the guts of cockroaches with a lignocellulosic diet were unclassified members of *Porphyromonadaceae* “Cluster V” (i.e., “Cockroach cluster” and “Termite cockroach cluster”).

## Discussion

In this study, we compared the physicochemical conditions and bacterial microbiota in the individual gut compartments of litter-feeding cockroaches. Our results confirm previous findings from wood-feeding and omnivorous species, which detected strong differences between foregut, midgut, and hindgut [26, 27, 30], and support the notion that abiotic and biotic conditions in the gut microenvironment are major drivers of bacterial community structure in cockroach guts. Our analysis of abundance and distribution of family-level lineages among five lignocellulose-feeding cockroach species revealed highest similarity among homologous gut compartments, particularly the hindgut. However, the proportion of core taxa shared between wood- and litter-feeding species was much higher in omnivorous cockroaches than in wood-feeding termites, which indicates that diet is not a major driver of community structure in cockroach guts. This conclusion is corroborated by the absence of lineages implicated in fiber digestion in wood-feeding termites from wood- and litter-feeding cockroaches.

### Differences in physicochemical conditions

Physicochemical conditions in the gut of litter-feeding cockroaches (this study) do not differ fundamentally from those in omnivorous and wood-feeding species [26–28, 30]. In all species investigated, the crop is moderately acidic, which has been attributed to a putative fermentation of ingested sugars by microorganisms already in the early studies of *Blattella germanica* and *Periplaneta americana* by Wigglesworth [40], and was later substantiated by the accumulation of lactate and acetate in this compartment [26, 27, 41]. This matches the large populations of lactic acid bacteria in the crop of *P. americana* documented by Kane and Breznak [41] and the predominance of *Streptococcaceae* or *Lactobacillaceae* in the crop of wood-feeding and litter-feeding cockroaches ([27], this study, Additional file 2: Table S1).

The increase in pH along the midgut, with a maximum in the posterior region, is most likely caused by host secretions, including the excretory fluid of the Malpighian tubules, whose nitrogenous components are expected to provide substantial buffering capacity [42]. It is not clear whether the low concentrations of lactate in midgut and hindgut are due to an absorption equilibrium between host and symbionts, as postulated for *Periplaneta americana* [43], or to a high turnover of the lactate pool, as demonstrated for lower termites [44, 45].

Although the major gut compartments of all cockroach species investigated to date are anoxic at the gut center ([26, 27, 30], this study), the redox potential of the gut contents differs substantially between species. The omnivorous *S. lateralis* [26], the litter-feeding *Byrsotria rothi* (this study), and the wood-feeding *Panesthia angustipennis* [27] show negative redox potentials (below − 100 to − 200 mV) at the center of all gut compartments (except for the rectum of *B. rothi*). In the litter-feeding *Ergaula capucina* and *Pycnoscelus surinamensis*, the redox potential was generally positive, decreasing from 150 to 300 mV in the crop to a range of 0 to 100 mV in the anterior hindgut.

The difference in redox profiles indicate differences in the redox-active metabolites in the respective compartments. Only two of the cockroach species examined to date, *Panesthia angustipennis* [27] and *Byrsotria rothi* (this study), accumulate hydrogen in the crop. This is in agreement with the negative redox potential of the crop observed in both species and the absence of hydrogen-consuming processes (viz., methanogenesis and reductive acetogenesis) in the crop of cockroaches [37]. By contrast, in the omnivorous cockroaches *Blaberus* sp. and *Shelfordella lateralis*, hydrogen accumulation is restricted to the midgut or anterior hindgut, reaching magnitudes of 29 and 24 kPa, respectively [26, 30]. In all cases, the hydrogen partial pressures observed in the respective compartments range between 20 and 30 kPa, which surpass the values reported for certain wood-feeding termites [45, 46].

As in omnivorous and wood-feeding species [26, 27, 30], hydrogen concentrations in the hindgut paunch of the litter-feeding species were close to or below the detection limit, which indicates that hydrogen-consuming processes (i.e., methanogenesis and homoacetogenesis) provide a strong hydrogen sink in all cockroaches investigated to date.

### Microenvironmental conditions determine community structure

The similarity of the microenvironmental conditions in the hindgut compartments of all cockroaches investigated to date matches the high similarity of the bacterial communities of most cockroach species ([31, 11], this study). Considering our observation that a major proportion of the total bacterial community in the hindgut of wood- and litter-feeding cockroaches consists of bacterial taxa that belong to bacterial families that are consistently represented in the hindgut of all species investigated (core families; Fig. 5), the hindgut compartment must provide essentially the same ecological niches for its microbiota, irrespective of the diet of the host.

By contrast, the bacterial communities in the crop and midgut of each wood-feeding and litter-feeding species differ substantially from that of the hindgut, showing highest similarity to the corresponding compartment in the same host (Figs. 3 and 5 [27];. Also in a detritivorous *Panchlora* sp. that lives in the refuse pile of leaf-cutter ants, each gut compartment harbors a distinct bacterial community that differs substantially from that of the fungal gardens or the waste material deposited by the ants [31]. The representation of bacterial lineages from the same core families in the homologous gut compartments of different cockroaches and the strong correspondence of certain bacterial lineages with specific physicochemical parameters are best explained by the stochastic uptake of bacteria from the environment and the subsequent selection and proliferation of certain bacterial lineages from the inoculum. The inoculation occurs either with the food or by coprophagy, which seems to be common among cockroaches [47–49] and is essential for the normal development of the first instar in *Blattella germanica* [50].

The gut microenvironment has been identified as a strong selective factor shaping the compartment-specific bacterial communities also in higher termites [20]. Despite ample opportunity for a vertical transmission of gut bacteria via proctodeal trophallaxis (feeding on the hindgut content of nestmates), even the termite gut microbiota comprises numerous bacterial lineages that were obviously acquired by horizontal transfer from other species or from the environment [51]. It remains to be investigated whether the highly abundant *Dysgonomonadaceae* (Bacteroidales termite cluster V), *Rikenellaceae,* and *Ruminococcaceae* are part of an ectosymbiotic community on intestinal thelastomatid nematodes (pinworms), as shown in *P. angustipennis* [52]. Overall, the presence of numerous gut-specific clades among the microbiota of both termites and cockroaches also supports the hypothesis that most microbial ecosystems are dominated by specialist taxa [53].

### Host diet and putative cellulose digestion

One major hypothesis on the assembly of intestinal communities concerns the role of the host diet, which should select for bacterial taxa specialized on the degradation of its recalcitrant constituents or the supplementation of deficient components. While different artificial diet regiments have been shown to change the hindgut community in the omnivorous *Blattella germanica* and the litter-feeding *Pycnoscelus surinamensis* [33, 54], no such effect has been observed in *Shelfordella lateralis* [35]. Also in *Periplaneta americana*, the core gut community appears to be stable and resilient to changes in diet [34]. Our results for litter-feeding cockroaches confirm the general similarity of the bacterial communities in the hindguts of all cockroaches and their difference from those in termites [11]. The slight differences between the hindgut communities of wood- and litter-feeding cockroaches and omnivorous species were much less pronounced than those between termites from different diet groups (Fig. 8).

The two most dominant phyla in the hindgut communities of wood- and litter-feeding cockroaches (*Firmicutes* and *Bacteroidetes*) predominate not only the gut communities of all cockroaches but also those of fungus-feeding termites [55]. This matches the surprisingly high similarity in the overall gut community structure between these only distantly related host groups, which has been explained by a convergent adaptation of the microbiota to the protein-rich diet common to cockroaches and macrotermitine termites [11]. The response of the hindgut community in *P. surinamensis* to a diet supplemented with different proportions of *Termitomyces* fungus supports this hypothesis [54].

The core families present in the hindgut of all lignocellulose-feeding cockroaches are abundantly represented (45–98%) among the bacterial communities of all cockroaches, irrespective of their feeding group, and even more abundant in omnivorous than litter-feeding species (Fig. 6b). Their low abundance in the hindgut of wood-feeding termites underscores that their presence is not determined by the lignocellulosic diet. Bacterial lineages representing the lignocellulolytic community associated with the wood fibers in higher termites, such as uncultured members of the “Treponema I” clade and the *Fibrobacteria* [10, 56], were not represented at all or encountered in low relative abundance only in the gut of *Ergaula capucina* (1% unclassified *Fibrobacterales*). However, it has been shown in the lower termite *Reticulitermes flavipes* that low-abundant bacteria in particular may drive diet-induced changes in gut community composition [57].

It is well documented that termites efficiently degrade the cellulose contained in their diet but hardly any of the lignin [58–60]. Except for the work on *Periplaneta americana* [61], such data is lacking for cockroaches. Although the litter-feeding cockroach species examined in this study were maintained on an entirely lignocellulosic diet, we observed that *Pycnoscelus surinamensis* prefers the softer leaf lamina over the more recalcitrant petiole and veins. Based on the similarity between the hindgut microbiota of panesthiine cockroaches (which dwell in decaying wood) and fungus-cultivating macrotermitine termites, it has been proposed that wood-feeding Panesthiinae digest wood-degrading fungi rather than the wood itself [27]. This implicates not only cellulose and hemicelluloses but also fungal proteins and other microbial biomass as important dietary components. Based on our present results, this hypothesis can be extended to litter-feeding cockroaches, which underscores the need to analyze the dietary components that are actually digested by members of the different feeding groups.

## Conclusion

The presence of closely related bacterial lineages in the hindgut of all cockroaches, irrespective of phylogenetic position or feeding group, strongly suggests that the gut habitat, rather than host diet, plays a critical role in constraining the structure of microbial communities in cockroaches. Future studies will have to describe further mechanisms of selection in the cockroach gut environment and assign functional roles to individual members of the gut microbial communities.

## Methods

### Sampling and dissection

Cockroaches of the species *Ergaula capucina* (Corydiidae, Corydiinae) (formerly Polyphagidae [62];, *Byrsotria fumigata* and *Byrsotria rothi* (Blaberidae, Blaberinae), and *Pycnoscelus surinamensis* (Blaberidae, Pycnoscelinae) were purchased from a commercial breeder (J. Bernhardt, Halsbrücke, Germany, http://www.schaben-spinnen.de). All colonies were then maintained in ventilated polypropylene containers (length 27 cm, width 20 cm, height 10 cm) continuously in the dark at 25 °C on the same diet of dried oak leaf litter and water for at least 2 months. In all cases, successful molting and maturation of freshly hatched cockroaches over several instars indicated that the colonies could be sustained on this particular diet. Only adult female insects were selected for the experiments.

### Microsensor measurements

To assess physicochemical conditions in each gut compartment, intestinal oxygen and hydrogen concentrations, pH, and redox potential were measured with microelectrodes (50-μm tip diameter; Unisense, Aarhus, Denmark). Oxygen and hydrogen microsensors were calibrated as described previously [22] using N_2_, synthetic air (21% O_2_), and a H_2_/N_2_ mixture (5% H_2_). The pH microelectrode was calibrated with commercial pH standard solutions (pH 4.0, 7.0, and 10.0). The redox microelectrode was calibrated with saturated solutions of quinhydrone in pH standards (pH 4.0 and 7.0). For pH and redox microelectrodes, the electric potential was measured against an Ag/AgCl reference electrode. For the measurements, the guts were dissected, placed in glass-faced chambers, fixed with insect pins to a bottom layer of silicone, and covered with air-saturated Insect Ringer’s solution (7.5 g NaCl, 0.35 g KCl, and 0.21 g CaCl_2_ per liter) [26]. It was not possible to obtain all parameters from the same gut preparation.

### Library construction

Cockroaches were dissected, and the guts were separated into crop, midgut, and hindgut compartments as previously described [26]. The gut compartments of three adult females of each species were placed separately in 2-ml tubes containing 750 μl sodium phosphate buffer (120 mM; pH 8.0) and homogenized with a polypropylene pestle. DNA was extracted and purified using a bead-beating protocol [63]. Extraction success was monitored by observing DNA integrity on an agarose gel. DNA quality was checked via spectrophotometric evaluation of absorption at 230, 260, and 280 nm (NanoDrop 1000 Spectrophotometer, Thermo Scientific, Waltham, USA) and fluorometric quantification (Qubit Fluorometer, Thermo Scientific, Waltham, USA). The V3-V4 region of the 16S rRNA genes was amplified using the universal bacterial primers 343Fmod and 784Rmod [46] and tagged with sample-specific hexameric barcodes [19]. Purified PCR products were normalized to equimolar amounts, pooled and commercially sequenced (2 × 350 nt paired-end sequencing) on an Illumina MiSeq platform (GATC Biotech, Konstanz, Germany).

The iTag libraries obtained in this study and previously published datasets obtained from termites [19, 13] were processed as previously described [36]. Briefly, paired-end reads with a minimum length of 250 bp and a maximum expected error of 0.5 were assembled into contigs and quality-trimmed (no homopolymers > 10 nucleotides, no ambiguities, average phred score > 25 on a moving window of five nucleotides), and the barcode and primer sequences were removed using *mothur* [64]. Sequences in each sample were clustered at a threshold of 99% similarity with *dnaclust* [65] and de-replicated and aligned with the *mothur* aligner. The original contigs (before quality trimming) of the samples obtained in this study were deposited in the sequence read archive (SRA) of the National Center for Biotechnology Information (NCBI, Bioproject PRJNA448568).

### Comparison of community structure

Aligned sequences were screened, degapped, and clustered into operational taxonomic units (OTUs) at 97% sequence similarity. OTU sampling coverage was estimated from rarefaction curves [66]. Expected richness [37], diversity [38], and evenness [39] of the communities were calculated for each sample. The OTUs were assigned to taxonomic groups using the Ribosomal Database Project (RDP) naïve Bayesian classifier implemented in *mothur* with a confidence threshold of 80% in combination with a manually curated reference database (DictDb v. 3.0; [36]). The libraries were subsampled to the size of the smallest sample (53,896 reads per sample for the comparison between the nine samples in this study; 1643 reads for the comparison between all hindgut communities from 28 hosts). Community structure was compared using the taxonomy-dependent Bray-Curtis metric (based on the classification results), a statistic used to quantify the compositional dissimilarity between two different samples, based on counts in each sample [67], and using the phylogeny-dependent weighted UniFrac algorithm [68] embedded in *mothur*. The high dimensionality of the pairwise dissimilarity scores was then compressed to two dimensions via non-metric multidimensional scaling (NMDS) using the *vegan* package in R [69]. Covariance between community structure, gut compartment, and physicochemical parameters were determined by permutational multivariate analysis of variance (PERMANOVA) and visualized by canonical correspondence analysis (CCA) using the *adonis* function, both implemented in the *vegan* package (for details, see Additional file 2: Table S2).

### Analysis of core microbial taxa

To identify core microbial lineages, all unclassified reads and all reads in taxa represented by fewer than ten reads were removed from the dataset. Bacterial genera that were present in at least 70% of all samples from a group of insect hosts or from a specific compartment were considered core genera of this group. For bacterial families, this threshold was set to 100%. The similarity on the family level between the gut communities of the five cockroach species with a lignocellulosic diet was determined using the *Morisita-Horn* index [70] and visualized using an arc diagram, implemented in the *vegan* and *arcdiagram* [71] packages in R, respectively.

## Supplementary information

**Additional file 1: Figure S1.** Species richness as function of sequence depth for the nine gut samples. Each curve represents the number of identified OTUs (97% sequence similarity) as a function of the number of sequenced reads after quality filtering. The vertical line indicates the minimum number of reads to which all samples were subsampled.

**Additional file 2: Table S1.** Interactive spreadsheet of the relative abundance of genus-level bacterial groups from all samples analysed in this study, based on subsamples of 1643 sequences per sample. The categories on the left allow switching between different taxonomic levels, to explore individual bacterial lineages. **Table S2.** Analysis of covariance of environmental variables selected for the canonical correspondence analysis (CCA) by permutational multivariate analysis of variance using distance matrices (PERMANOVA, Anderson 2001, implemented in the function *adonis* of the *vegan* package in R). Columns indicate (Sums) sums of squares, (F) model strength, (R2) squared correlation coefficient, and (p) probability value with marks for *p* ≤ 0.001 (***), *p* ≤ 0.01 (**). **Table S3.** Relative abundance of core bacterial families in the amplicon libraries of crop, midgut and hindgut of five cockroaches with a lignocellulosic diet. The core status was defined by the consistent presence in the respective compartment in all host species [Ec, Ergaula capucina; Bf, Byrsotria fumigata; Ps, *Pycnoscelus surinamensis* (litter-feeding, this study); Pa, Panesthia angustipennis; Se, Salganea esakii (wood-feeding, Bauer et al. [27])]. **Table S4.** Bacterial core taxa across cockroaches and termites from different feeding guilds. This Excel spreadsheet contains the 18 core bacterial families in the lignocellulose-feeding cockroaches. Listed are the relative abundances of the bacterial genera within these families across cockroaches and termites from different feeding guilds. **Table S5.** Information about the insect species used in this study, their diet, and the origin of the datasets included in the analyses.

## Data Availability

The sequence datasets generated and analyzed during the current study are available in the Sequence Read Archive (SRA) of the National Center for Biotechnology Information (NCBI), https://www.ncbi.nlm.nih.gov/bioproject/PRJNA448568/.
