# Supplementary material for: Diet is not the primary driver of bacterial community structure in the gut of litter-feeding cockroaches
Source: BMC Microbiol. 2019 Oct 30;19:238. doi: 10.1186/s12866-019-1601-9 (PMC6864750; doi:10.1186/s12866-019-1601-9)
Supplement: Supplementary file 1 — Additional file 1: Figure S1. Species richness as function of sequence depth for the nine gut samples. Each curve represents the number of identified OTUs (97% sequence similarity) as a function of the number of sequenced reads after quality filtering. The vertical line indicates the minimum number of reads to which all samples were subsampled. [file 12866_2019_1601_MOESM1_ESM.pdf]

Supplementary Figures

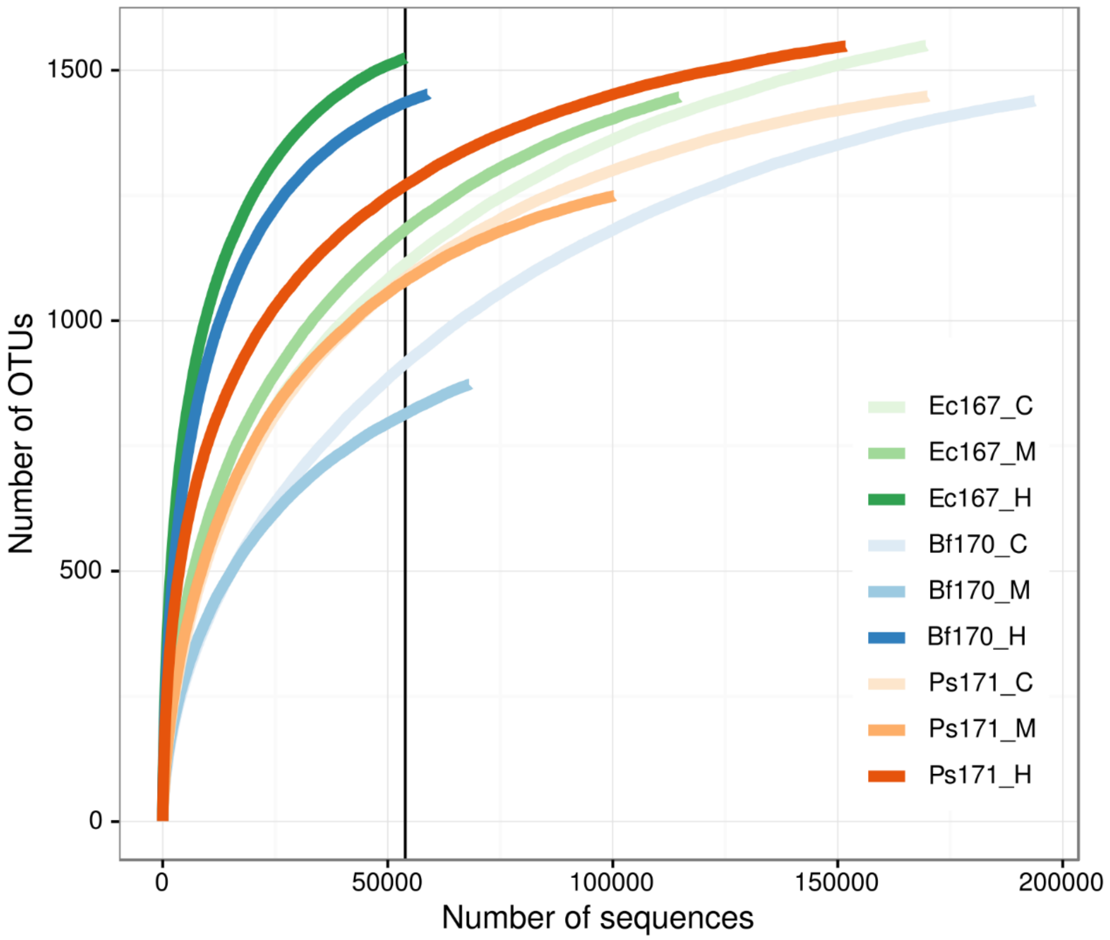

Figure S1 | Species richness as function of sequence depth for the nine gut samples. Each curve represents the number of identified OTUs (97% sequence similarity) as a function of the number of sequenced reads after quality filtering. The vertical line indicates the minimum number of reads to which all samples were subsampled.
